# Inner Ear Malformation Masquerading as CSF Rhinorrhea - New Perspectives of Management and Literature Review

**DOI:** 10.22038/IJORL.2023.70032.3385

**Published:** 2023-07

**Authors:** Nidhin Das K, Vidhu Sharma, Sarbesh Tiwari, Amit Goyal

**Affiliations:** 1 *Department of Otorhinolaryngology, All India Institute of Medical Sciences, Jodhpur, India.*; 2 *Department of Diagnostic and Interventional Radiology, All India Institute of Medical Sciences, Jodhpur, India.*

**Keywords:** Cochlear Implants, CSF leak, Inner ear malformation

## Abstract

**Introduction:**

Spontaneous cerebrospinal fluid (CSF) rhinorrhea is rare and may develop secondary to inner ear malformation. A possible diagnosis of CSF leak should be considered in any Pediatric patient who presents with hearing impairment, rhinorrhea, or otorrhea.

**Case Report:**

We describe a case of 13 months male infant presenting with rhinorrhoea which on evaluation found to be CSF oto-rhinorrhoea due congenital inner ear malformation. Imaging showed malformed inner ear on both sides with CSF leak on left side with bilateral profound sensory neural hearing loss. A multidisciplinary management was considered. Child underwent CSF leak repair on left side followed by Cochlear implantation on right side in another setting.

**Conclusion:**

This case is a perfect example to describe the cumbersome management of CSF leak with inner ear anomaly addressing the auditory habilitation on the grounds of recent innovations. As per available literature inner ear anomaly is an important subgroup of population of cochlear implant candidates with promising auditory outcomes.

## Introduction

Osseous labyrinthine malformations are fairly rare anomalies, representing approximately 20% of the cases of congenital sensorineural hearing loss (1). Rest 80% are contributed by membranous labyrinth anomalies which are occurring at cellular level. Here the bony architecture is essentially normal. There is spectrum of clinical presentation for inner ear anomaly (IEA) from speech language delay to intermittent watery nasal discharge. 

As complex is this disorder, that complex is its management part. In the beginning era of cochlear implantation (CI) one of the contraindications for the surgery was IEA. However soon in 1983 Mangaberia-Albernaz reported the first CI in IEA which was a Mondini deformity (2). 

Here we are discussing a peculiar case of inner ear malformation with an odd presentation, diagnosis, management and (re)habilitation. 

## Case Report

**Fig 1:**
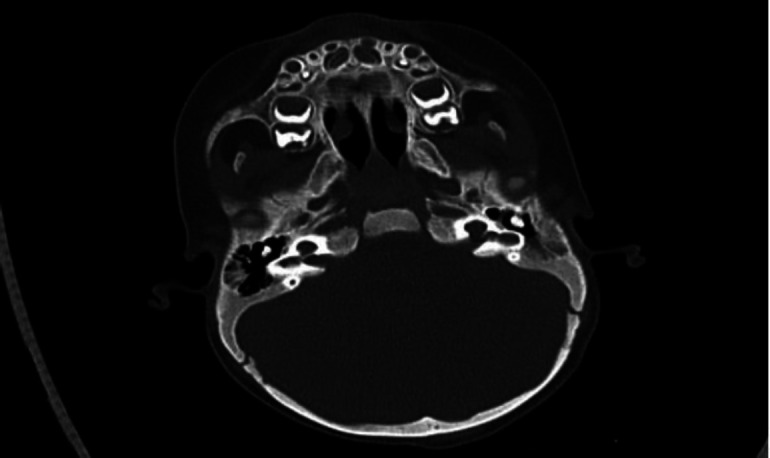
High resolution computed tomography temporal bones showing malformation of inner ear bilaterally (marked by black arrows)

**Fig 2:**
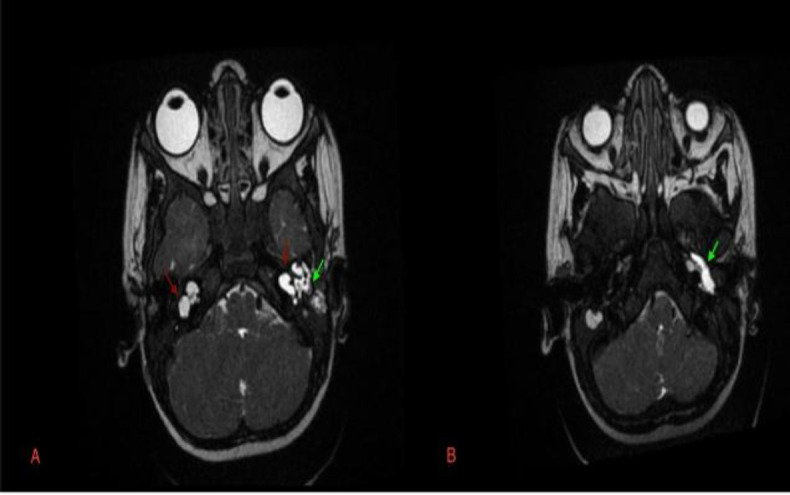
T2 Magnetic resonance images axial section showing bilateral inner ear malformation (red arrows) with CSF leaking to middle ear on left side (B) CSF seems extending from middle ear to eustachian tube on left side (green arrows)

A 13-month-old boy who had no previous history of head injury or trauma was brought by parents with chief complaints of spontaneous continuous watery nasal discharge from both sides since the age of 6 months. Parents also noticed poor responses to sound. He had no recent history of upper respiratory illness, and no history of acute otitis media or otorrhea. On clinical examination, child was not responding to loud sounds and rest of the ear, nose and throat examination was normal. He was subsequently admitted, audiology and imaging was planned. Auditory brainstem response (ABR) testing showed bilateral profound sensorineural hearing loss. The acoustic immittance (AI) test revealed a “C” shaped curve on the left and an “A” shaped curve on the right. CT and MRI scanning of the temporal bones demonstrated left side wide internal acoustic canal (IAC) with absent cribriform plate (Figure 1&2).

Cystic cochlea was seen without any internal architecture. Vestibule was visualized separately from cochlea with figure of eight configuration- likely type-1 incomplete partition of cochlea. Lateral and posterior semicircular canals were not visualized. Left cochlear segment of VIII nerve complex was hypoplastic. 

**Fig 3:**
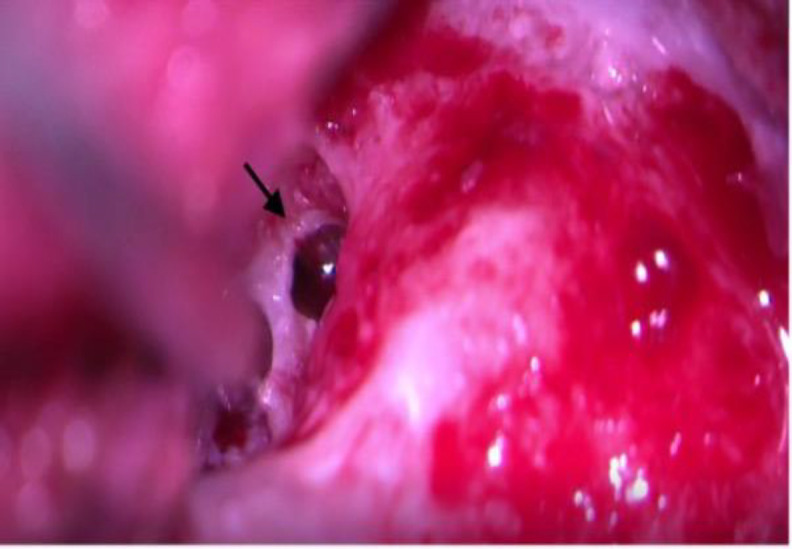
Intra operative image of CSF leak repair showing CSF leak from oval window on left side (black arrow)

Fluid signal was seen in the left mesotympanum and mastoid cavity with extension along the left Eustachian tube. On right side IAC was mildly widened, and a cribriform plate was present. Cochlea was cystic with absent modiolus. Vestibule & semicircular canals were visualized normally. The cisternal portions of the right VII-VIII nerve complex were seen normally. Child underwent left side radical mastoidectomy with CSF leak repair (Figure 3). 

**Fig 4:**
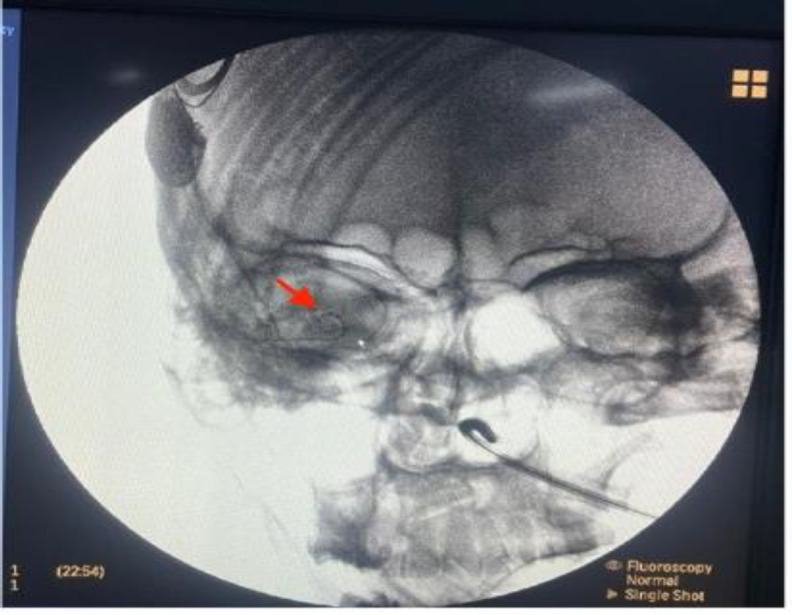
Post cochlear implantation C Arm generated X-ray showing the implant on right side (red arrow)

Intraoperatively CSF leak was found to be from both round window and oval window. Child recovered uneventfully. After the recommended period of habilitation with hearing aid he underwent right side cochlear implantation by Veria technique via cochleostomy (Figure 4). 

Switch on was done on post operative day 21 and auditory verbal therapy was started.

## Discussion

Inner ear anomaly masquerading as CSF rhinorrhoea is always a fascinating entity for ENT surgeons. So is the management of inner ear anomaly from the perspective of complication as well as (re) habilitation.

Radical mastoidectomy is defined as canal wall down procedure with removal of the ossicles, stripping of middle ear mucosa, plugging of eustachian tube with or without cul de sac closure of external auditory canal. 

This procedure in a case of CSF otorrhea especially with inner ear anomaly has been a controversy (3). There always exists a dispute of permanent loss of hearing by air conduction against life threatening meningitis due to CSF leak. A significant rate of recurrent meningitis has been reported in post repair patients especially in a concealed space like middle ear. Also, there are chances of post repair intracranial epithelial inclusions and mucocele formation. All the osseous labyrinthine anomalies appreciable on radiology are usually named as Mondini deformity. However, the true Mondini dysplasia rarely presents with CSF leak. Sennaragulu et al stated the true Mondini deformity as Incomplete partition type II (IP-II) in which cochlea consists of 1.5 turns, in which the middle and apical turns coalesce to form a cystic apex, accompanied by a dilated vestibule and enlarged vestibular aqueduct (VA)(4). Common route of CSF leak in cochlear dysplasia is internal acoustic meatus in which cribriform plate is usually absent. Tympanomeningeal hiatus (Hyrtle’s fissure), aqueduct of cochlea, inferior tympanic canaliculus (Jacobson’s canal) are the other common routes of CSF leak.

Radiology is both diagnostic and treatment deciding. Here the question to be answered is which modality to choose. In a case of suspected inner ear anomaly presenting as CSF leak both HRCT temporal bones and MRI are to be considered as they will help to classify the anomaly as well as associated other anomaly such as hypoplastic VIII^th^ nerve or aberrant course of VII^th^ nerve (2).

As one can see in the classifications of inner ear anomaly (IEA) there are varieties of malformation of cochlea with considerable architectural differences. Hence one should choose the appropriate electrode as per standard recommendation. Electrodes with complete contact ring are preferred in common cavity, IP I & III. All kind of electrodes can be used in IP II and Large vestibular aqueduct syndrome, while short electrodes are used in Cochlear hypoplasia (2). A special electrode array was described by Beltrame et al. (2005) for common cavity anomaly which has a non-active tip which inserted through a superior labyrinthotomy and taken out through second labyrinthotomy which is made 4mm below the first one and hooked to the other end (5).

Common intraoperative challenges faced are CSF gusher and facial nerve anomaly. Facial nerve has a complex temporal bone course and the deciding factor for the final position of the facial nerve is the inner ear development. Preoperative radiology and intraoperative facial nerve monitoring are the steps to reduce the facial nerve injury.

CSF gusher is defined as the egress of profuse clear fluid upon making an opening in inner ear. This is common in CI surgery for IEA. Phelps et al classified CSF gusher into oozing (gentle flow of clear fluid and gusher (profuse flow) (6). A small cochleostomy is recommended allowing the electrode array to partly block the CSF leak which is further reinforced with fat, facia and/or fibrin glue. Post operative continuous lumbar drain can be considered. Other intra operative manoeuvres to enhance the ease of electrode insertion are putting the patient in reverse Trendelenburg position or waiting for 15 minutes during which the flow will reduce. As a final option subtotal petrosectomy is also explained in literature. Despite substantial success associated with CI, outcomes in paediatric CI recipients with IEA are variable. In a study conducted by Isaiah A et al, they found Cochlear dysplasia, vestibular dysplasia and cochlear nerve hypoplasia were associated with poor speech recognition, although the outcomes in children with enlarged vestibular aqueduct were similar to those of children with normal inner ear anatomy (65%) (7).

## Conclusion

Inner ear anomaly can be masquerade as CSF rhinorrhoea. These individuals are considered to be a unique subgroup of candidates in CI surgery. It is important to follow a single classification system when dealing with IEA in order to standardise the treatment, follow up and (re)habilitation. Both MRI and CT should be considered during the work up of such patients and two important intraoperative challenges are aberrant facial nerve course and CSF gusher.
